# Is early-morning class exposure associated with lower positive mental health among university students? Conditional associations and heterogeneous profiles

**DOI:** 10.3389/fpubh.2026.1951005

**Published:** 2026-09-24

**Authors:** Wei Yang, Jili Meshage, Ri-Zhenmo Hu, Xiulan Jiang

**Affiliations:** 1 School of Economics, Management and Tourism, Xichang University, Xichang, Sichuan, China; 2 School of Teacher Education, Xichang University, Xichang, Sichuan, China; 3 Department of Preschool Education, Xichang Minzu Preschool Normal College, Xichang, Sichuan, China

**Keywords:** bedtime procrastination, early-morning class exposure, emotion-regulation difficulties, latent profile analysis, positive mental health, social jetlag

## Abstract

**Background:**

University is a period of heightened vulnerability for student mental health, yet institutional schedules remain understudied as a modifiable determinant. Drawing on social zeitgeber theory, we tested whether early-morning class exposure is associated with positive mental health through social jetlag and emotion-regulation difficulties, whether bedtime procrastination conditions this association, and whether students' risk configurations are heterogeneous.

**Methods:**

Data were obtained from 1,437 university students in Sichuan and Yunnan, China. Early-morning class exposure was operationalized as the number of weekdays on which the first class began at or before 08:00. Using partial least squares structural equation modeling with 5,000 bootstrap resamples, we tested direct, indirect, and moderated serial indirect associations while controlling for demographic covariates. Latent profile analysis then identified configurations of these variables, with positive mental health compared across profiles as a distal outcome.

**Results:**

More frequent early-morning class exposure was associated with lower positive mental health (total effect β = –0.270, 95% CI [–0.316, –0.221]). This association operated partly through a serial indirect association: greater exposure predicted more social jetlag, which predicted more emotion-regulation difficulties. Emotion-regulation difficulties alone did not mediate the association independently. Bedtime procrastination amplified this process, strengthening the exposure-jetlag link at higher levels of procrastination (interaction β = 0.451, 95% CI [0.413, 0.489]). Latent profile analysis identified five distinct risk configurations: students combining frequent classes with stable sleep and effective emotion regulation reported the highest positive mental health, whereas those with co-occurring emotion-regulation difficulties and elevated schedule risk reported the lowest.

**Conclusions:**

These findings extend social zeitgeber theory to institutional course scheduling, showing that early-morning classes relate to students' positive mental health through a sequential temporal-psychological process that bedtime procrastination can intensify. Supporting student mental health may require attending jointly to institutional schedule design and individual sleep regulation, pending confirmation from longitudinal and objective-data designs.

## 1 Introduction

Positive mental health, comprising positive affect, effective psychological functioning, and satisfying social relationships (1–3), is a central concern during the university transition and predicts subsequent health (4, 5). Sleep quality and regular routines are closely linked to this positive mental health (6–9), yet research has emphasized individual habits over the institutional timing that structures them.

Course timing is one such institutional factor, and its consequences are well documented. Morning classes are associated with earlier waking, shorter sleep, lower attendance, and poorer performance (10), and objective sleep measures relate to academic functioning (11). Later starts show the reverse pattern, with longer sleep, lower social jetlag, and better daytime functioning (12, 13). Because sleep regularity differs from duration (14), the recurrence of early classes across the week, rather than sleep loss on any single night, may be what matters for students. We conceptualize early-class exposure as a recurring institutional timing demand over which students have limited control.

Social Zeitgeber Theory explains why such a demand should matter. External schedules that conflict with biological rhythms produce social jetlag, the difference between study-day and free-day sleep timing (15–18). Social jetlag is associated with poorer quality of life and mental health (19, 20) and poorer cognitive and academic performance (21), and chronotype and sleep quality are similarly associated with depression and suicidal ideation among university students (22). Whether early-class exposure itself reaches positive mental health through social jetlag remains untested. Emotion-regulation difficulties may complete this sequence. Insufficient sleep can impair emotion recognition and self-control (23), consistent with broader links between sleep problems, emotion-regulation difficulties, depression, and anxiety (24, 25). This sequence need not operate uniformly, however, because students differ in whether they advance bedtime before early classes. Bedtime procrastination, a sleep-specific self-regulatory failure (26), is associated with psychological distress and poorer sleep (27, 28) and may condition how strongly early classes translate into social jetlag.

A variable-centered model can estimate these average direct, indirect, and conditional associations, but it does not reveal how the same factors combine within individual students. Early-morning class exposure, social jetlag, bedtime procrastination, and emotion-regulation difficulties may not occur independently or uniformly; instead, they may cluster into recurring configurations characterized by different levels of risk. Person-centered approaches are designed to identify such heterogeneity (29), and latent profile studies in related sleep and health-behavior domains have identified distinct configurations associated with different levels of mental health among university students (30, 31). Whether the four focal factors in the present study form distinct within-student configurations, and whether positive mental health differs across these configurations, has not been established.

The present study therefore combined structural equation modeling with latent profile analysis in a sample of Chinese university students. The structural model examined the direct association between early-morning class exposure and positive mental health, specific and serial indirect associations through social jetlag and emotion-regulation difficulties, and whether bedtime procrastination moderated the association between early-morning class exposure and social jetlag and the corresponding serial indirect association. Latent profile analysis then identified recurring configurations of early-morning class exposure, social jetlag, bedtime procrastination, and emotion-regulation difficulties, and compared positive mental health across the resulting profiles. Together, these analyses examine early-morning classes as a potentially modifiable institutional timing exposure, extend Social Zeitgeber Theory to the context of higher education, and evaluate both population-average associations and heterogeneous institutional-behavioral risk configurations. Figure 1 summarizes the theoretical framework and main findings of this study.

**Figure 1:**
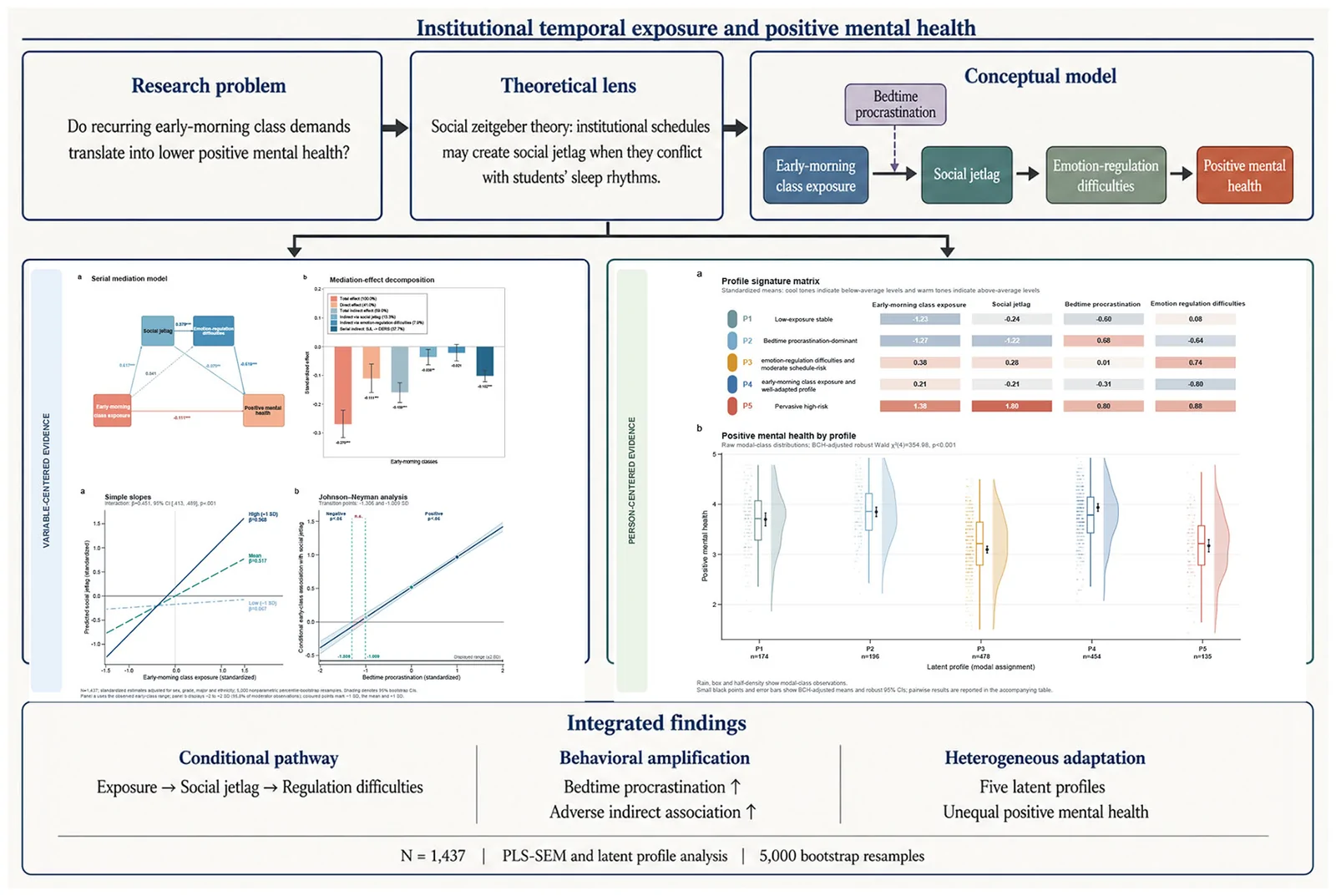
Conceptual framework of the study.

## 2 Theoretical framework and hypotheses

### 2.1 Social Zeitgeber Theory

Social Zeitgeber Theory explains how external social arrangements contribute to the synchronization and regulation of individual biological rhythms. The theory proposes that biological rhythms are shaped not only by physical cues, such as natural light, but also by socially structured activities. The timing of work, social interactions, and daily activities provides important social cues that regulate the sleep-wake rhythm. Ehlers et al. (15) initially proposed that social events may destabilize biological rhythms by disrupting daily routines and social time cues, thereby increasing vulnerability to mood disturbances. Grandin et al. (16) further suggested that changes in social rhythms, disruptions in biological rhythms, and affective states may form a continuous process. Social Zeitgeber Theory therefore identifies a fundamental mechanism through which social institutions become embedded in biological and psychological regulation. Institutions may influence behavior not only by determining what individuals do, but also by determining when they act.

Previous research has primarily examined social zeitgebers in the context of cue loss, stressful life events, and disruptions to daily routines. This literature has emphasized whether social time cues are stable and regular. However, the stability of a social time cue does not necessarily imply that it is adaptive. A social timing requirement may be persistent, regular, and predictable while remaining misaligned with an individual's intrinsic biological rhythm. Research on social jetlag indicates that conflicts between work or study schedules and preferred sleep timing produce systematic differences between scheduled days and free days (17, 18). To explain stable but potentially maladaptive institutional timing requirements, we distinguish between the regularity and temporal alignment of social zeitgebers. Regularity refers to the stability and predictability of a social time structure. Temporal alignment refers to whether its timing is compatible with an individual's intrinsic biological rhythm.

This distinction provides an important perspective for understanding university course schedules. Course schedules do more than organize teaching activities. They repeatedly determine when students wake, commute, enter the classroom, and begin cognitively demanding activities. Course schedules therefore constitute one of the most stable and restrictive social zeitgebers in university life. The significance of early-morning classes lies not only in their early start time. They also impose a recurring, institutionally determined timing requirement on students with diverse sleep rhythms and limited short-term opportunities to avoid that requirement.

Accordingly, we conceptualize early-morning class exposure as an institutionally imposed temporal exposure. It is an external timing requirement embedded in students' daily lives that constrains sleep-wake timing by requiring activity at an early hour. Students must typically wake earlier on days with early-morning classes. If their bedtime does not advance correspondingly, their schedules on class days and free days may gradually diverge, resulting in social jetlag. When this temporal misalignment repeatedly occurs, its implications extend beyond changes in sleep duration. Students must also maintain daytime psychological functioning despite persistent misalignment between social and biological time. This process may be associated with greater emotion-regulation difficulties and, in turn, with lower positive mental health.

Institutional timing requirements, however, do not necessarily produce identical consequences for all students. Early-morning classes determine when students must wake, but they cannot determine when students fall asleep on the preceding night. Bedtime procrastination therefore represents a critical behavioral link between institutional timing requirements and personal sleep schedules. It may be associated with the extent to which an externally imposed early wake time corresponds to greater social jetlag.

Social Zeitgeber Theory provides the overarching framework for the present study. We organize the proposed model as a sequential process linking institutional temporal structure, temporal alignment, psychological regulation, and mental-health outcomes. The purpose is not simply to characterize early-morning classes as an unfavorable scheduling practice. Instead, the model explains how institutionally determined timing becomes embedded in students' biological rhythms and psychological regulatory processes.

### 2.2 Early-morning class exposure and positive mental health

Institutional timing requirements may influence students' positive psychological functioning through several interrelated forms of daily strain. Early-morning classes require students to wake earlier, although university students often do not advance their bedtimes accordingly. Analyses of digital behavior and activity records indicate that morning classes primarily shorten nocturnal sleep by advancing wake times. Students generally do not fall asleep correspondingly earlier before these classes (10). A meta-analysis of school start times similarly found that earlier start times were generally associated with shorter sleep and more negative affect (12). Under flexible course schedules, students slept longer and reported better overall feelings, classroom motivation, and attentiveness on days when classes began later (32). These findings indicate that the impact of early-morning classes may reach beyond the immediate loss of sleep on any given night. Repeated early wake requirements may reduce recovery time, increase daytime fatigue, and deplete resources needed for positive experiences in learning, relationships, and personal development. A longitudinal meta-analysis found that better sleep predicted greater subsequent positive well-being, although the relationship may be bidirectional (6). Research on chronotype also suggests that sleep quality and resilience may link chronotype with mental-health symptoms (33). When early-morning classes recur throughout the week, their cumulative timing demands may therefore reduce students' emotional and social well-being.

**H1: Among university students, more frequent early-morning class exposure is linked to lower positive mental health**.

### 2.3 The mediating role of social jetlag

Social jetlag is defined as the difference in sleep midpoint between work/study days and free days. It represents a discrepancy between social and biological time (17, 18). Within an institutional social zeitgeber framework, early-morning classes primarily constrain students' wake times on class days. When students revert to their preferred sleep schedules on free days, the gap in sleep midpoint between class days and free days widens. Existing evidence identifies course timing as an important external source of social jetlag. University students generally wake earlier before morning classes but rarely advance their bedtimes to the same extent. This pattern results in sleep restriction and an earlier midpoint of sleep on class days (10). A large Norwegian study found that later school start times were associated with longer sleep on school days and lower social jetlag (13). A systematic review and meta-analysis similarly showed that school start times and the social clock jointly shape sleep duration, chronotype, and social jetlag among adolescents (34). Research on the transition to adulthood further suggests that sleep health and circadian misalignment influence the relationship between chronotype and lifestyle (35). Social jetlag therefore cannot be attributed entirely to personal sleep preferences. It also reflects the temporal configuration of institutional schedules.

Social jetlag has similarly been associated with poorer positive psychological functioning. Longitudinal research indicates that persistent or newly emerging social jetlag predicts subsequent depressive symptoms, daytime sleepiness, and lower quality of life (20). Among university students, greater social jetlag has also been associated with lower quality of life (19). Recent evidence further suggests that social jetlag and chronotype may connect physical symptoms with mental health (36). Students repeatedly switch between institutional time on class days and personal time on free days. This recurring transition may diminish energy, positive affect, and daily functioning, thereby reducing positive mental health.

**H2: Social jetlag mediates the negative association between early-morning class exposure and positive mental health**.

### 2.4 The mediating role of emotion-regulation difficulties

Emotion-regulation difficulties involve problems identifying, understanding, and accepting emotions. They also include difficulties controlling impulses, maintaining goal-directed behavior, and accessing effective strategies during emotional arousal. Institutional timing requirements may create persistent differences between weekday and weekend rhythms. They may also affect emotion regulation through acute sleep restriction, morning sleepiness, and inadequate recovery. Even without the cumulative influence represented by social jetlag, early-morning classes may impair sleep on the preceding night and functioning on the following day. These acute strains may increase emotion-regulation difficulties.

Sleep provides an essential foundation for effective emotion regulation. An integrative review concluded that insufficient sleep impairs the ability to identify emotional problems, select regulatory strategies, and implement those strategies effectively (23). A recent meta-analysis further identified a consistent association between insomnia symptoms and emotion-regulation difficulties (25). Research involving students also indicates that poor sleep quality may increase emotion-regulation difficulties through impaired daytime functioning and reduced self-control (37). This evidence supports the possibility that acute sleep loss associated with early-morning classes may translate into difficulties with psychological regulation.

Emotion-regulation difficulties may subsequently limit the ability to maintain positive affect, purpose, social connectedness, and effective daily functioning. Individual differences in emotion-regulation strategies are closely related to affective experiences, interpersonal relationships, and wellbeing (38). A latent profile study among university students found that those with broadly elevated emotion-regulation difficulties reported the lowest subjective wellbeing (39). Other studies have similarly linked emotion regulation with the mental health of university students (40, 41). Emotion-regulation difficulties may therefore constitute a separate indirect association between early-morning class exposure and poorer positive mental health.

**H3: Emotion-regulation difficulties mediate the negative association between early-morning class exposure and positive mental health**.

### 2.5 The serial mediating roles of social jetlag and emotion-regulation difficulties

Within the social zeitgeber framework, institutional schedules may influence mental health through a process that translates temporal maladaptation into psychological maladaptation. Early-morning classes advance wake and activity times on class days. However, students may not adjust their bedtimes or free-day schedules accordingly. The resulting social jetlag is not merely a difference in sleep duration. It also requires students to switch repeatedly between two temporal schedules. They follow institutionally determined social time on class days and return to sleep timing closer to their biological rhythms on free days. As this switching recurs, a temporary scheduling discomfort may develop into persistent rhythmic strain.

This rhythmic strain may subsequently impair the cognitive and behavioral resources required for emotion regulation. Longitudinal research indicates that persistent social jetlag predicts subsequent daytime sleepiness, depressive symptoms, and lower quality of life (20). Effective emotion regulation requires sustained attention, impulse control, and goal-directed behavior during emotional arousal. Recurrent misalignment in sleep-wake timing and the accompanying lack of recovery may weaken these capacities. Research on sleep and emotion regulation also suggests that sleep disturbances and emotion-regulation difficulties are not isolated conditions. Instead, both may contribute to the development of mental-health risks (23–25).

When individuals cannot effectively identify, understand, and regulate their emotions, the consequences of social jetlag may extend beyond transient fatigue and mood fluctuations. They may develop into persistent reductions in positive affect, psychological functioning, and social functioning. Social jetlag may therefore connect institutional timing requirements with psychological regulation. Emotion-regulation difficulties may subsequently transmit this rhythmic misalignment to positive mental health. Early-morning class exposure may thus be associated with poorer positive mental health through sequential increases in social jetlag and emotion-regulation difficulties.

**H4: Social jetlag and emotion-regulation difficulties serially mediate the negative association between early-morning class exposure and positive mental health**.

### 2.6 The moderating role of bedtime procrastination and the moderated serial mediation model

The constraints imposed by early-morning classes on student sleep schedules are inherently asymmetrical. By specifying attendance times, course schedules determine the latest time at which students can wake. However, students retain discretion over when they end their evening activities and go to bed. Because wake times are institutionally constrained while bedtimes remain behaviorally determined, the same early-morning schedule may not produce the same degree of social jetlag. Students who adjust their bedtimes to accommodate the next morning's schedule may achieve a relatively complete advance in their class-day sleep rhythm. Without a corresponding change in bedtime, the adjustment occurs primarily through earlier waking. This pattern restricts sleep on class days and increases the discrepancy between class-day and free-day schedules.

Bedtime procrastination occurs precisely at the point that institutional schedules do not directly control. It is conceptualized as the inability to go to bed at the desired time, even in the absence of external constraints. It is commonly conceptualized as a failure of self-regulation in the sleep context (26). Students generally adjust to morning classes by waking earlier rather than falling asleep earlier (10). This pattern indicates that an institutional requirement to wake early does not necessarily produce a corresponding advance in bedtime.

Research among Chinese university students found that bedtime procrastination linked a later chronotype with poorer sleep quality (42). Diary research also identified daily covariation among stress, bedtime procrastination, and sleep outcomes on the same night (43). Problematic smartphone use may additionally relate to sleep problems through mechanisms that include bedtime procrastination (44, 45). A recent meta-analysis found a consistent positive association between bedtime procrastination and psychological distress among university students (27). These findings suggest that bedtime procrastination is not merely a risk behavior associated with poor sleep. It may influence whether external timing requirements can be coordinated with personal sleep rhythms.

When bedtime procrastination is low, students may be more likely to adjust their evening activities and bedtimes before early-morning classes. Personal sleep schedules may therefore accommodate the institutionally imposed requirement to wake early. As bedtime procrastination increases, coordinating bedtime with the required wake time becomes more difficult. Early-morning classes are then more likely to produce sleep restriction and divergent rhythms between class days and free days. The positive association between early-morning class exposure and social jetlag may therefore become stronger as bedtime procrastination increases.

**H5: Bedtime procrastination positively moderates the association between early-morning class exposure and social jetlag**.

The implications of bedtime procrastination may extend beyond social jetlag itself. The extent to which early-morning class exposure corresponds to social jetlag determines the degree of temporal misalignment at the outset of the subsequent psychological process. At higher levels of bedtime procrastination, equivalent exposure may produce greater social jetlag. This misalignment may subsequently contribute to greater emotion-regulation difficulties and poorer positive mental health. In contrast, effective adjustment of bedtime behavior may buffer the conflict between early-morning classes and personal sleep schedules. It may consequently weaken the transmission of this association through subsequent psychological processes. Bedtime procrastination may therefore modify both the initial relationship and the entire serial indirect association.

**H6: Bedtime procrastination moderates the serial indirect association between early-morning class exposure and positive mental health through social jetlag and emotion-regulation difficulties**.

## 3 Methods

### 3.1 Participants and procedure

The survey was administered across universities in Sichuan and Yunnan provinces from May to July 2026. Participation was entirely voluntary, and all participants read the study description and consent form prior to completing the survey. All data were collected anonymously, and no identifying information was recorded.

Of the 1,609 returned questionnaires, 172 were excluded according to predefined quality standards, yielding a final sample of 1,437 valid responses (effective response rate: 89.3%). The final sample comprised 569 male and 868 female students, including 1,032 lower-year and 405 upper-year students (see Table 1). The dataset contained no missing values or duplicate records.

**Table 1:** Demographic characteristics of the sample.

Variable	Category	*n*	%
Sex	Male	569	39.6
	Female	868	60.4
Year of study	Lower year	1,032	71.8
	Upper year	405	28.2
Field of study	Liberal arts	928	64.6
	Sciences	509	35.4
Ethnicity	Han	1,022	71.1
	Ethnic minority	415	28.9

*N* = 1, 437. Percentages were calculated within each demographic variable.

Except for the positive mental health measure, which referred to experiences during the previous month, all schedule-related questions referred to the most recent complete teaching week of the current semester.

### 3.2 Measures

#### 3.2.1 Early-morning class exposure

Early-morning class exposure was measured as the number of days on which participants attended an early-morning class during the most recent complete teaching week. For each weekday from Monday to Friday, the first scheduled class was coded 1 if it began at or before 8:00 a.m. and 0 otherwise. The five daily scores were totaled to generate a weekly exposure score ranging from 0 to 5. This operationalization is consistent with previous research using course start times to characterize daily timing demands among university students (10).

#### 3.2.2 Social jetlag

Social jetlag was calculated using the core timing indicators of the Munich Chronotype Questionnaire (17, 46). Participants provided separate reports of their sleep-onset and wake times for class days vs. free days, from which the sleep midpoint was derived for each day type. Social jetlag was defined as the absolute difference between the sleep midpoint on free days and class days, expressed in hours. Greater values indicated more pronounced discrepancies between personal biological rhythms and socially imposed schedules.

#### 3.2.3 Emotion-regulation difficulties

Emotion-regulation difficulties were assessed using the 16-item Difficulties in Emotion Regulation Scale (47). Items were scored on a 1-to-5 scale, where higher scores reflected more difficulties in emotional awareness, acceptance, and regulation.

#### 3.2.4 Positive mental health

The 14-item Mental Health Continuum-Short Form was used to assess positive mental health (1, 48). Participants indicated the frequency with which they had experienced each state over the preceding month. Items were scored on a 1-to-5 scale, where higher scores reflected greater emotional, psychological, and social wellbeing.

#### 3.2.5 Bedtime procrastination

The nine-item Bedtime Procrastination Scale (26) was administered to assess bedtime procrastination. All items were scored on a 1-to-5 scale, with Items 2, 3, 7, and 9 reverse-coded. Higher scores reflected a greater inclination to postpone bedtime even when no external obstacles were present.

#### 3.2.6 Covariates

All structural models controlled for sex, year of study, field of study, and ethnicity. Sex was dummy-coded as male = 0 and female = 1; year of study as lower year = 0 and upper year = 1; field of study as liberal arts = 0 and sciences = 1; and ethnicity as Han = 0 and ethnic minority = 1.

PLS-SEM was selected as the primary variable-centered approach because the study pursued an explanatory-predictive objective. Alongside estimating the theoretically specified direct, specific indirect, serial indirect, and conditional indirect associations, we assessed the explained variance and cross-validated predictive relevance of the endogenous constructs using R^2^ and Q^2^ predict generated through PLSpredict (49–51). PLS-SEM also provided a unified bootstrap framework for estimating the interaction and corresponding conditional indirect associations. CB-SEM would also have been feasible given the large sample and reflective constructs; therefore, the choice of PLS-SEM reflected the study's additional predictive objective rather than any presumed inability of CB-SEM to estimate the proposed model. The measurement structure was assessed separately using a correlated three-factor CFA before structural estimation.

Analyses were conducted in R 4.6.0. After evaluating measurement quality and assessing potential common method bias, we used PLS-SEM with 5,000 bootstrap samples to estimate the direct, indirect, and conditional indirect associations. LPA was conducted separately using early-class exposure, social jetlag, bedtime procrastination, and emotion-regulation difficulties as the four profile indicators. Positive mental health was subsequently compared across profiles as a distal outcome using the BCH procedure.

## 4 Results

### 4.1 Descriptive statistics and correlations

Table 2 presents, students attended early-morning classes on an average of 2.486 days per week, and their mean social jetlag was 1.560 h. Early-morning class exposure showed positive correlations with social jetlag and emotion-regulation difficulties, and a negative correlation with positive mental health. Social jetlag showed a positive relationship with emotion-regulation difficulties, and both variables were negatively associated with positive mental health. Bedtime procrastination was positively associated with social jetlag and emotion-regulation difficulties, while its correlation with positive mental health was weakly negative.

**Table 2:** Descriptive statistics and correlations among the study variables.

Variable	*M*	*SD*	1	2	3	4	5
1. Early-morning class exposure	2.486	1.677	1.000				
2. Social jetlag	1.560	0.855	0.510^***^	1.000			
3. Emotion-regulation difficulties	32.544	10.118	0.237^***^	0.401^***^	1.000		
4. Positive mental health	49.186	8.891	–0.269^***^	–0.336^***^	–0.576^***^	1.000	
5. Bedtime procrastination	26.916	7.725	–0.010	0.161^***^	0.079^**^	–0.054^*^	1.000

*N* = 1, 437. Positive mental health was represented by a positively scored total score. ^*^p < 0.05, ^**^p < 0.01, ^***^p < 0.001.

### 4.2 Measurement quality, common method bias, and model quality

The three constructs demonstrated satisfactory reliability and convergent validity. Across the three constructs, Cronbach's α ranged from 0.874 to 0.918, McDonald's ω_*t*_ from 0.877 to 0.918, and composite reliability from 0.877 to 0.918. The average variance extracted ranged from 0.554 to 0.705, and all standardized factor loadings exceeded 0.700, ranging from 0.713 to 0.872. The correlated three-factor measurement model demonstrated excellent fit, χ^2^/df = 1.058, CFI = 0.999, TLI = 0.999, RMSEA = 0.006, 90% CI [0.000, 0.015], and SRMR = 0.014.

Discriminant validity was assessed using the Fornell–Larcker criterion and the heterotrait–monotrait ratio of correlations (HTMT). The square roots of AVE (reported in Table 3) were 0.782 for emotion-regulation difficulties, 0.840 for positive mental health, and 0.744 for bedtime procrastination. Each value exceeded the absolute correlations between the corresponding construct and the other latent constructs (Table 2). The HTMT values ranged from 0.064 to 0.651, all below the conservative threshold of 0.850. These findings jointly supported adequate discriminant validity among the three constructs.

**Table 3:** Reliability and convergent validity of the multi-item constructs.

Construct	α	ω_*t*_	Loading range	CR	AVE	$\sqrt{\text{AVE}}$
Emotion-regulation difficulties	0.885	0.887	0.713–0.845	0.887	0.611	0.782
Positive mental health	0.874	0.877	0.778–0.872	0.877	0.705	0.840
Bedtime procrastination	0.918	0.918	0.729–0.758	0.918	0.554	0.744

ω_*t*_ denotes total McDonald's ω; CR, composite reliability; AVE, average variance extracted, $\sqrt{\text{AVE}}$ is the square root of AVE, used as the Fornell–Larcker diagonal for discriminant-validity.

Potential common method bias was examined using three complementary procedures. The first factor extracted in Harman's single-factor test accounted for 22.0% of the total variance. The single-factor confirmatory model exhibited poor fit, CFI = 0.369, TLI = 0.279, RMSEA = 0.220, and SRMR = 0.253, while the maximum full-collinearity VIF was 1.615. Taken together, these diagnostics indicated no severe common method bias in the present data, although such statistical procedures cannot completely rule out shared method variance

The structural model also demonstrated satisfactory fit, with an SRMR of 0.019 and an NFI of 0.984. The maximum inner VIF was 1.531, indicating no problematic structural collinearity. The model explained 48.6% of the variance in social jetlag, 16.3% in emotion-regulation difficulties, and 35.8% in positive mental health. Q^2^ predict values ranged from 0.038 to 0.453, supporting the predictive relevance of the endogenous constructs.

### 4.3 Direct and indirect effects

As shown in Table 4, after controlling for demographic covariates, early-morning class exposure was positively linked to social jetlag, β = 0.517, 95% CI [0.478, 0.554], *p* < 0.001. Social jetlag was positively associated with emotion-regulation difficulties, β = 0.379, 95% CI [0.324, 0.434], *p* < 0.001. Emotion-regulation difficulties showed a negative relationship with positive mental health, β = –0.519, 95% CI [–0.559, –0.478], *p* < 0.001. Early-morning class exposure maintained a significant negative direct association with positive mental health, β = –0.111, 95% CI [–0.160, –0.060], *p* < 0.001. H1 was thus supported.

**Table 4:** Path coefficients and indirect effects.

Path or effect	β	95% CI	*p*
Early-morning class exposure → Social jetlag	0.517	[0.478, 0.554]	<0.001
Early-morning class exposure → Emotion-regulation difficulties	0.041	[–0.015, 0.095]	0.146
Early-morning class exposure → Positive mental health	–0.111	[–0.160, –0.060]	<0.001
Social jetlag → Emotion-regulation difficulties	0.379	[0.324, 0.434]	<0.001
Social jetlag → Positive mental health	–0.070	[–0.122, –0.018]	0.005
Emotion-regulation difficulties → Positive mental health	–0.519	[–0.559, –0.478]	<0.001
Early-morning class exposure → Social jetlag → Positive mental health	–0.036	[–0.063, –0.010]	0.005
Early-morning class exposure → Emotion-regulation difficulties → Positive mental health	–0.021	[–0.050, 0.008]	0.146
Early-morning class exposure → Social jetlag → Emotion-regulation difficulties → Positive mental health	–0.102	[–0.122, –0.083]	<0.001

The indirect association between early-morning class exposure and positive mental health through social jetlag was significant, β = –0.036, 95% CI [–0.063, –0.010], *p* = 0.005, supporting H2. The separate indirect association through emotion-regulation difficulties was not significant, β = –0.021, 95% CI [–0.050, 0.008], *p* = 0.146. H3 was therefore not supported. The serial indirect association through social jetlag and emotion-regulation difficulties was significant, β = –0.102, 95% CI [–0.122, –0.083], *p* < 0.001, supporting H4.

Figure 2 further presents the relative contributions of the indirect effects. Figure 2a shows that early-morning class exposure was positively associated with social jetlag, which was positively associated with emotion-regulation difficulties. Emotion-regulation difficulties were, in turn, negatively associated with positive mental health. The direct path from early-morning class exposure to emotion-regulation difficulties was not significant, consistent with the lack of support for H3. As indicated in Figure 2b, the total indirect effect represented 59.0% of the total effect. The serial indirect effect through social jetlag and emotion-regulation difficulties accounted for 37.7% of the total effect. This proportion exceeded the separate indirect effect through social jetlag, which accounted for 13.3%, and the separate indirect effect through emotion-regulation difficulties, which accounted for 7.9%. The association between early-morning class exposure and positive mental health was therefore primarily represented by a serial indirect association involving social jetlag and emotion-regulation difficulties.

**Figure 2:**
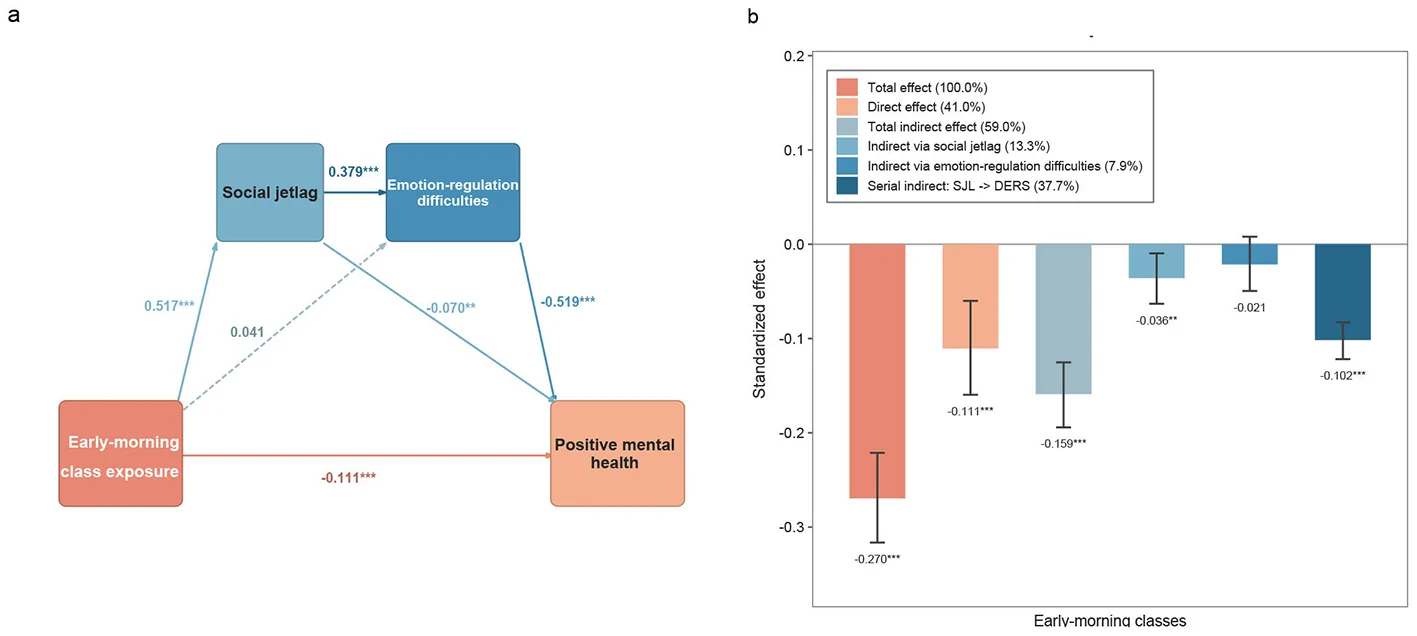
Structural associations linking early-morning class exposure, social jetlag, emotion-regulation difficulties, and positive mental health. **(a)** Serial mediation model. **(b)** Mediation-effect decomposition. ^*^p < 0.05, ^**^p < 0.01, ^***^p < 0.001.

### 4.4 Moderation and moderated serial mediation

As shown in Table 5, the interaction between early-morning class exposure and bedtime procrastination was positively associated with social jetlag, β = 0.451, 95% CI [0.413, 0.489], *p* < 0.001. Simple-slope analyses showed that the positive association was weaker at low bedtime procrastination, β = 0.067, 95% CI [0.004, 0.128], *p* = 0.036. The association was stronger at the mean level, β = 0.517, 95% CI [0.478, 0.554], *p* < 0.001, and strongest at high bedtime procrastination, β = 0.968, 95% CI [0.926, 1.007], *p* < 0.001. H5 was therefore supported.

**Table 5:** Moderation and moderated mediation effects.

Test	β	95% CI	*p*
Moderation			
Bedtime procrastination → Social jetlag	0.173	[0.130, 0.215]	<0.001
Early-morning class exposure × Bedtime procrastination → Social jetlag	0.451	[0.413, 0.489]	<0.001
Low bedtime procrastination (−1 SD):	0.067	[0.004, 0.128]	0.036
Mean bedtime procrastination:	0.517	[0.478, 0.554]	<0.001
High bedtime procrastination (+1 SD):	0.968	[0.926, 1.007]	<0.001
Moderated serial mediation			
Low bedtime procrastination (−1 SD):	−0.013	[−0.026, −0.001]	0.036
Mean bedtime procrastination:	−0.102	[−0.122, −0.083]	<0.001
High bedtime procrastination (+1 SD):	−0.190	[−0.226, −0.157]	<0.001
Index of moderated serial mediation	−0.089	[−0.106, −0.072]	<0.001

Figure 3 further illustrates the moderation pattern. As shown in Figure 3a, the positive slope relating early-morning class exposure to social jetlag increased markedly with bedtime procrastination. The association was weak at low bedtime procrastination, stronger at the mean, and strongest at high bedtime procrastination. The Johnson-Neyman analysis further identified the regions of significance. Early-morning class exposure was negatively associated with social jetlag when bedtime procrastination was below –1.306 SD. The association was not significant between –1.306 and –1.009 SD. Above –1.009 SD, the association became significantly positive. Thus, higher bedtime procrastination strengthened the positive association between early-morning class exposure and social jetlag, consistent with H5.

**Figure 3:**
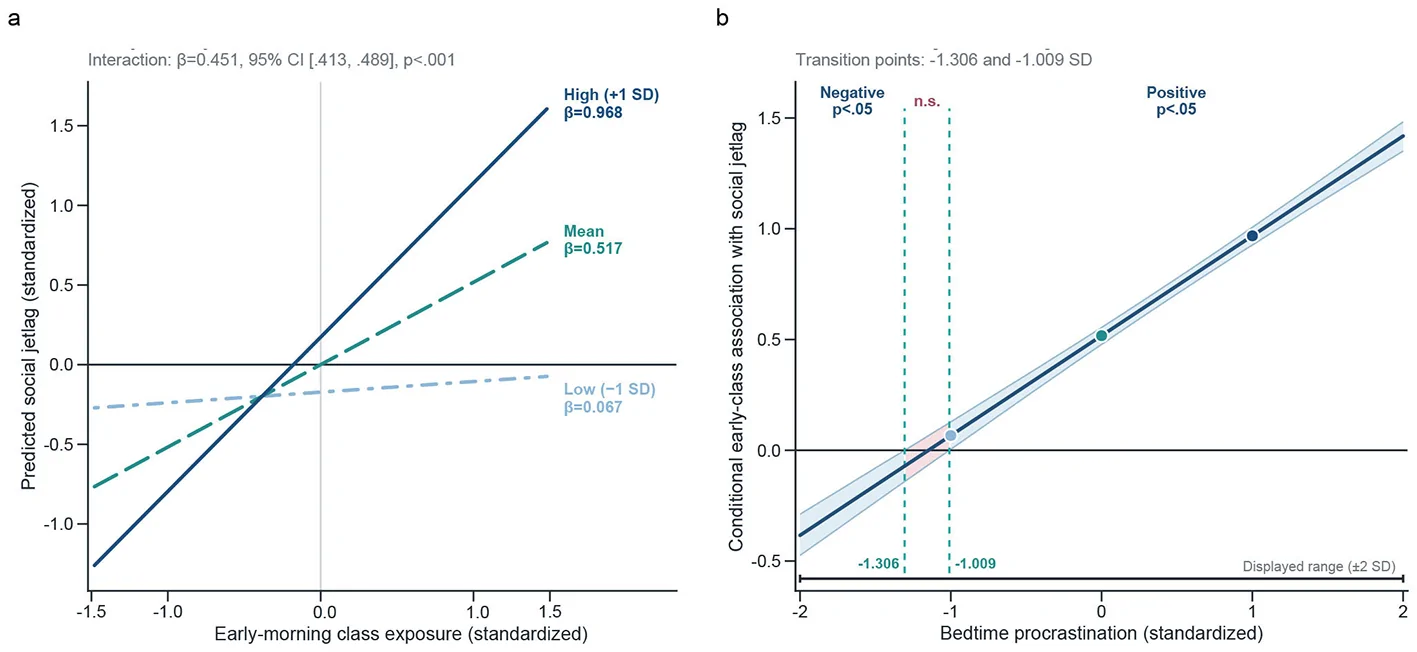
Moderating role of bedtime procrastination and conditional serial indirect effects. *N* = 1, 437; standardized estimates adjusted for sex, grade, major, and ethnicity; 5,000 nonparametric percentile-bootstrap resamples. Shading denotes 95% bootstrap confidence intervals. **(a)** Simple slopes. **(b)** Johnson–Neyman analysis.

The conditional indirect effects followed the same gradient. At low, mean, and high levels of bedtime procrastination, the serial indirect associations through social jetlag and emotion-regulation difficulties were –0.013, –0.102, and –0.190, respectively. The corresponding 95% confidence intervals were [–0.026, –0.001], [–0.122, –0.083], and [–0.226, –0.157]. The index of moderated serial mediation was –0.089, 95% CI [–0.106, –0.072], *p* < 0.001, supporting H6. At the mean level of bedtime procrastination, the total indirect effect was –0.159, 95% CI [–0.194, –0.125], and the total effect was -0.270, 95% CI [–0.316, –0.221].

### 4.5 Latent profile model and profile characteristics

Table 6 compares the fit of the one- through six-profile solutions. AIC and SABIC decreased continuously as the number of profiles increased, whereas BIC reached its minimum at the five-profile solution and increased after a sixth profile was added. The 500-replication parametric bootstrap likelihood ratio test (BLRT) was significant at every comparison step (*p* < 0.002), including the four-versus-five-profile and five-versus-six-profile comparisons. However, adding a sixth profile reduced entropy from 0.691 to 0.671, lowered the mean maximum posterior probability from 0.788 to 0.765, and reduced the smallest profile from 9.4% to 8.9%, with limited additional improvement. Considering BIC, classification quality, profile size, parsimony, and theoretical interpretability together, the five-profile solution was retained.

**Table 6:** Comparison of latent profile model fit indices.

Profiles	AIC	BIC	SABIC	Entropy	Mean maximum posterior probability	Smallest profile	BLRT *p*
2	15,612.5	15,702.1	15,648.1	0.745	0.927	39.3%	<0.002
3	15,181.9	15,318.9	15,236.3	0.778	0.897	16.4%	<0.002
4	15,050.3	15,234.7	15,123.6	0.658	0.801	17.0%	<0.002
**5**	**14,990.8**	**15,222.6**	**15,082.9**	**0.691**	**0.788**	**9.4%**	**<0.002**
6	14,958.4	15,237.7	15,069.4	0.671	0.765	8.9%	<0.002

BLRT used 500 parametric bootstrap samples; therefore, the smallest reportable *p* value was <0.002. The bold row indicates the retained solution.

For this solution, posterior assignment probabilities across profiles ranged from 0.700 to 0.859, and the odds of correct classification (52) ranged from 6.150 to 58.940, all exceeding the conventional threshold of 5; the smallest profile comprised 135 students (9.4%). No single, universally agreed cutoff exists for entropy. Published guidance describes values near 0.700 as generally acceptable and values below approximately 0.600 are unusual to see published (53). The present entropy value is close to this common benchmark, and the same methodological review already cited above (29) illustrates its recommended procedure with an entropy of 0.690, nearly identical to the value obtained here, describing it as reflecting acceptable classification certainty. As a complementary index, the mean maximum posterior probability declined smoothly and monotonically from the two-profile to the six-profile solution (0.927 to 0.765), without the non-monotonic dip observed in the entropy sequence between the three- and four-profile solutions, indicating that classification certainty followed an expected pattern as profiles increased rather than an anomalous one. Together with the OCC values, the monotonicity of the mean maximum posterior probability, profile size, and the entropy value's position within this accepted range, these results support the adequacy of the five-profile classification.

Figure 4a presents the standardized indicator means for the five profiles. Profile 1, the low-exposure stable profile (*n* = 174, 12.1%), was characterized by low early-morning class exposure and low bedtime procrastination, while social jetlag and emotion-regulation difficulties remained close to the sample mean. Profile 2, the bedtime-procrastination-dominant profile (*n* = 196, 13.6%), showed relatively low early-morning class exposure and social jetlag but elevated bedtime procrastination. Profile 3, the emotion-regulation difficulties and moderate schedule-risk profile (*n* = 478, 33.3%), showed slightly elevated early-morning class exposure and social jetlag, together with distinctly elevated emotion-regulation difficulties. Profile 4, the early-morning class exposure and well-adapted profile (*n* = 454, 31.6%), was characterized by approximately average early-morning class exposure, low social jetlag and bedtime procrastination, and the lowest level of emotion-regulation difficulties. Profile 5, the pervasive high-risk profile (*n* = 135, 9.4%), showed above-average levels across all four indicators, with social jetlag being particularly elevated.

**Figure 4:**
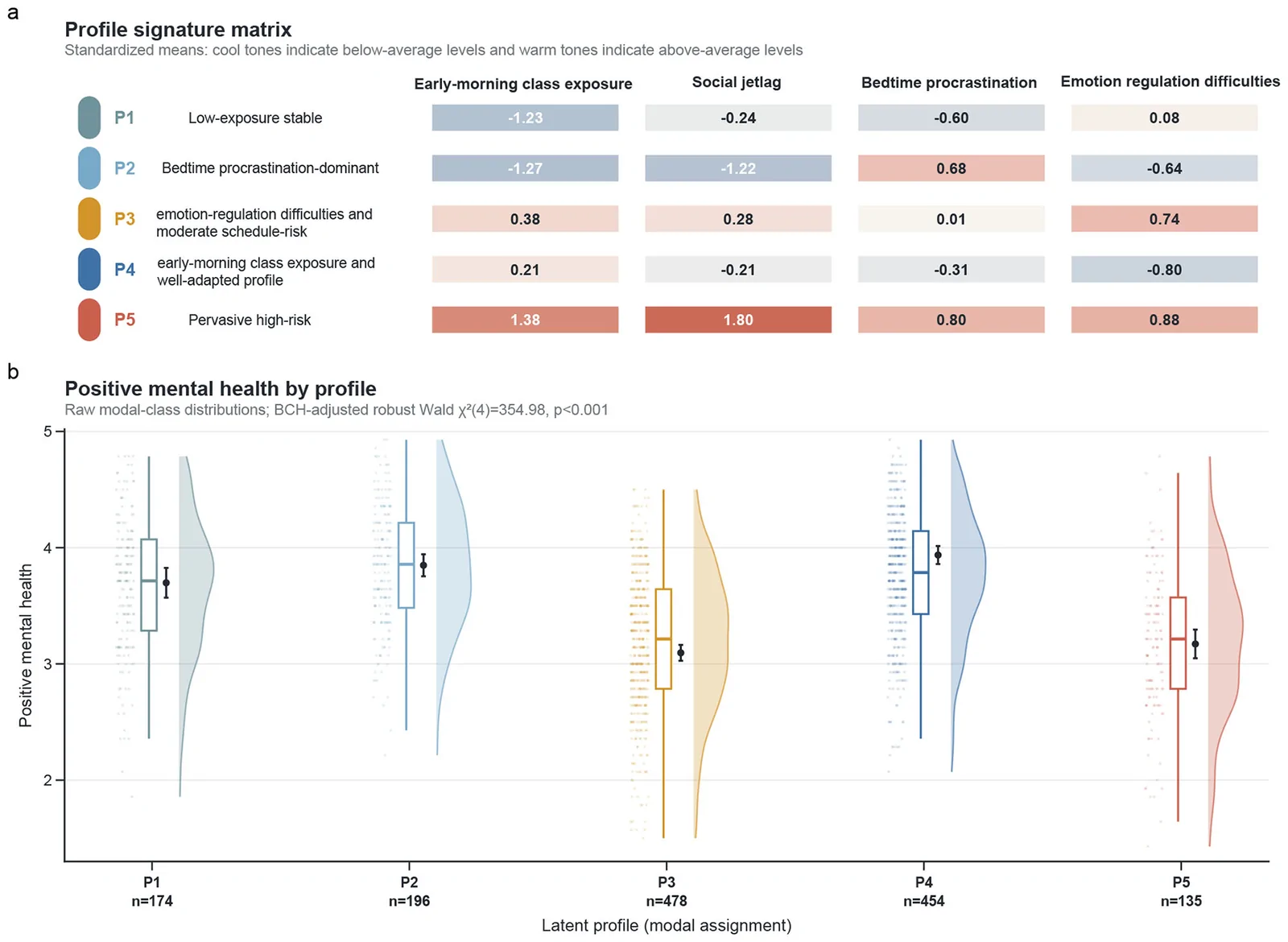
Five latent profiles of university students' schedules and emotion regulation. **(a)** Standardized means of early-morning class exposure, social jetlag, bedtime procrastination, and emotion-regulation difficulties across the five profiles. **(b)** Raw distributions and BCH-adjusted means of positive mental health across profiles. Black points and error bars represent BCH-adjusted means and robust 95% confidence intervals, respectively.

### 4.6 Differences in positive mental health across profiles

Positive mental health was treated as a distal outcome and compared across the five profiles using the BCH method. The overall difference was statistically significant, Wald χ^2^ (4) = 354.984, *p* < 0.001. As shown in Table 7, the early-morning class exposure and well-adapted profile (P4) had the highest adjusted mean of positive mental health, followed by the bedtime-procrastination-dominant profile (P2). Following Holm correction, the difference between P4 and P2 was not statistically significant (*p* = 0.346). The low-exposure stable profile (P1) showed significantly lower positive mental health compared to P4, with a mean difference of –0.239, 95% CI [–0.397, –0.081], Holm-adjusted *p* = 0.012. The emotion-regulation difficulties and moderate schedule-risk profile (P3) and the pervasive high-risk profile (P5) exhibited the lowest adjusted means, and the difference between these two profiles did not achieve statistical significance (*p* = 0.346).

**Table 7:** BCH comparison of positive mental health across the five latent profiles.

Profile	BCH mean	Robust SE	95% CI	Group
P1 Low-exposure stable	3.698	0.065	[3.570, 3.826]	b
P2 Bedtime-procrastination dominant	3.849	0.048	[3.754, 3.944]	ab
P3 Emotion-regulation difficulties and moderate schedule risk	3.095	0.035	[3.027, 3.163]	c
P4 Early-morning class exposure and well-adapted	3.937	0.040	[3.859, 4.014]	a
P5 Pervasive high-risk	3.171	0.063	[3.048, 3.295]	c

Positive mental health was represented by the MHC-SF item mean, with higher scores indicating better mental health.

Figure 4b displays the raw distributions of positive mental health together with the BCH-adjusted means and robust 95% confidence intervals for the five profiles. Although the distributions overlapped across profiles, a clear overall pattern was observed: P4 showed the highest positive mental health, followed by P2 and P1, whereas P3 and P5 showed substantially lower levels. The exact BCH-adjusted means, robust standard errors, confidence intervals, and Holm-adjusted significance groupings are reported in Table 7.

Profile membership did not differ significantly by sex, year of study, field of study, or ethnicity, with all *p* values ≥ 0.123. Taken together, these findings indicate that positive mental health did not vary solely according to the level of early-morning class exposure. Instead, it differed across configurations of early-morning class exposure, social jetlag, bedtime procrastination, and emotion-regulation difficulties.

## 5 Discussion and conclusions

This study conceptualized early-morning classes as a form of institutional temporal exposure and combined structural equation modeling with latent profile analysis to examine their associations with positive mental health among university students. The results indicated that early-morning class exposure was associated with positive mental health through a serial indirect association involving social jetlag and emotion-regulation difficulties, and that bedtime procrastination amplified this association. The findings also revealed substantial heterogeneity in students' configurations of temporal behaviors and psychological regulation.

### 5.1 Associations through social jetlag and emotion regulation

Early-morning class exposure was associated with lower positive mental health. This finding extends previous research beyond sleep, attendance, and academic performance to encompass positive psychological functioning. Earlier studies have linked early-morning classes or training schedules to earlier wake times, shorter sleep duration, and poorer academic performance (10, 54). Our findings additionally indicate that class timing may be linked to students' emotional, social, and psychological well-being. Because positive mental health reflects individuals' capacity to live positively and function effectively, this result broadens the range of outcomes considered in research on early-morning classes (4).

The mediating role of social jetlag identifies a proximal association through which institutional time demands may be associated with individual psychological functioning. Early-morning classes require students to wake earlier, but their bedtimes may not shift correspondingly. This discrepancy may create a misalignment between socially imposed schedules and individual sleep rhythms. Previous research has linked circadian instability and social jetlag to poorer mental health (55, 56). Our results further indicated that this temporal misalignment was linked to greater emotion-regulation difficulties, which in turn were related to lower positive mental health. This pattern is consistent with evidence that sleep disruption may impair attentional control, inhibitory capacity, and emotional stability (25, 37), as well as with research linking emotion regulation closely to mental health (24, 57).

The direct path linking early-morning class exposure to emotion-regulation difficulties did not reach statistical significance. This result indicates that emotion-regulation difficulties may not arise directly from exposure to early-morning classes. Instead, they may become more apparent in conjunction with persistent temporal misalignment associated with class schedules. Social jetlag may therefore represent a key correlate linking institutional time demands with difficulties in psychological regulation.

### 5.2 Bedtime procrastination as a behavioral boundary condition

Bedtime procrastination strengthened the association between early-morning class exposure and social jetlag, as well as the serial indirect association with positive mental health. Early-morning classes primarily determine when students must wake the following day, whereas whether they advance their bedtime accordingly depends partly on the regulation of their bedtime behavior. Previous studies have associated bedtime procrastination with insufficient sleep, daytime fatigue, and psychological distress (27, 58, 59). The current study expands upon this evidence by indicating that bedtime procrastination serves not only as an independent sleep-related risk behavior but also as a factor shaping the degree to which institutional time demands correspond to social jetlag. When fixed early wake-time requirements coincide with delayed bedtimes, temporal misalignment and its associated psychological correlates may become more pronounced. However, the weak association or reverse trend observed at very low levels of bedtime procrastination should be interpreted cautiously and does not indicate that early-morning classes have a protective effect.

### 5.3 Heterogeneity in temporal behaviors and psychological regulation

The latent profile analysis showed that early-morning class exposure, social jetlag, bedtime procrastination, and emotion-regulation difficulties did not occur together in a uniform manner. Students in the early-morning class exposure and well-adapted profile maintained relatively high positive mental health despite having comparatively frequent early-morning classes. This finding suggests that early-morning classes may not have the same implications for all students. Their associations with positive mental health may depend on whether students are able to maintain relatively stable sleep schedules and effective emotion regulation.

By contrast, students in the emotion-regulation difficulties and moderate schedule-risk profile and the pervasive high-risk profile reported lower positive mental health, whereas those in the bedtime-procrastination-dominant profile did not show the lowest levels. This pattern suggests that a single risk factor may be insufficient to determine an individual's overall mental health status. Instead, the co-occurrence of emotion-regulation difficulties and multiple temporal risks may warrant greater attention. This finding is consistent with profile-based research on sleep, emotion regulation, and lifestyle behaviors among university students (30, 39, 60, 61). Whereas the structural equation model identified average associations across the overall sample, the latent profile analysis captured distinct configurations of risk factors at the individual level. The two analytical approaches therefore provided complementary perspectives.

This heterogeneity is directly illustrated by the present data: although the bedtime-procrastination-dominant profile (P2) showed the second-highest level of bedtime procrastination among the five profiles, it was not associated with the lowest positive mental health. The lowest levels of positive mental health were observed only when emotion-regulation difficulties co-occurred with elevated schedule-related risk (P3) or when risk was pervasive across all four indicators (P5). This pattern cannot be recovered from the variable-centered model's average associations, which cannot indicate whether a given risk factor's association with mental health depends on which other risk factors are simultaneously present. This is the specific analytic question that motivated the inclusion of latent profile analysis in this study.

### 5.4 Theoretical and educational implications

This study has three principal theoretical contributions. First, it reconceptualizes early-morning classes from a routine feature of class schedules into a form of institutional temporal exposure that repeatedly constrains students' daily routines. Second, it extends social zeitgeber theory to the context of university class scheduling and identifies social jetlag as a proximal correlate linking class timing to positive mental health. Third, the moderating role of bedtime procrastination suggests that temporal misalignment is jointly shaped by institutional arrangements and individual behavior. It should therefore not be attributed exclusively either to class scheduling or to students' self-discipline.

From an educational perspective, universities should consider the cumulative weekly frequency and consecutive scheduling of early-morning classes instead of simply eliminating all such classes. Class-schedule optimization could also be combined with sleep-health education to help students reduce bedtime procrastination and maintain regular sleep schedules. Students who experience both substantial social jetlag and emotion-regulation difficulties may require more targeted support in time management and psychological regulation. The latent profile findings further suggest that identifying students at elevated risk should not be based solely on the number of early-morning classes they attend. Their temporal behaviors and psychological-regulation characteristics should also be considered.

### 5.5 Limitations and future directions

Several limitations deserve to be mentioned. First, the cross-sectional and self-reported data do not allow the temporal ordering of the study variables to be firmly established. Future research could examine the proposed associations using repeated assessments, natural experiments involving changes in class schedules, and objective or intensive longitudinal measures such as wearable devices, sleep diaries, and ecological momentary assessment.

Second, this study did not include an independent measure of chronotype, such as a preference-based morningness–eveningness scale. Previous research has shown that individual differences in chronotype are associated with sleep timing and mental health outcomes (22, 33). Therefore, the associations involving social jetlag observed in the present study may have been partly influenced by unmeasured differences in chronotype. Future studies should incorporate an independent measure of chronotype, ideally together with objective sleep data, to better disentangle the relative contributions of institutionally imposed timing and individual circadian preference.

Third, early-morning class exposure was assessed mainly by the weekly count of days on which students attended such classes. This measure did not distinguish between compulsory and elective classes, consecutive scheduling, commuting time, or actual attendance. Future studies should also account for potentially relevant factors such as sleep quality, daytime sleepiness, and academic stress. Fourth, the latent profile structure may depend on the characteristics of the sample and the indicators included in the analysis. Replication across different regions and universities is therefore needed. Longitudinal latent transition analysis could further determine whether students move between profiles as their class schedules and sleep routines change over time.

## Data Availability

The datasets generated and analyzed during the current study are available from the corresponding author upon reasonable request.
